# Leveraging Inter-Annotator Disagreement for Semi-Supervised Segmentation

**DOI:** 10.1109/ISBI53787.2023.10230706

**Published:** 2023-09-01

**Authors:** A. Sauer, M. Dmitrieva, H. Han, J. Rittscher

**Affiliations:** Department of Engineering Science, University of Oxford, Oxford, UK

**Keywords:** Image Segmentation, Multiple Annotations, Uncertainty Estimation, Semi-Supervised Segmentation

## Abstract

The low signal-to-noise ratio typically found in biomedical images often leads experts to disagree about the underlying ground truth segmentation. While existing approaches for multiple annotations try to resolve conflicting annotations, we instead focus on efficiently using pixels of disagreement to estimate areas of high uncertainty in the data and exploit this information for semi-supervised segmentation.

Pseudo-labelling approaches, which utilise unlabelled data by trying to match their own predictions, need to distinguish reliable from unreliable predictions. We propose to identify unreliable pseudo-labels from the output of a separate network that is trained to predict the uncertainty in the data based on conflicting annotations from different annotators.

Compared to other uncertainty estimation techniques like MC-Dropout or ensembling approaches, our approach has the two key advantages that its estimate stems directly from the data and that it is computationally more efficient. Using two public datasets, we show the effectiveness of our approach. ^1^

## 1 Introduction

The segmentation of biomedical images poses a challenging task since it often combines little annotated training data with a high level of noise and uncertainty compared to natural images. This higher degree of uncertainty is commonly manifested in significant inter-annotator variability and a growing numbers of approaches investigate how to model and handle this setting [1, 2, 3, 4]. These techniques mostly focus on the challenges associated with high inter-annotator disagreement like estimating the underlying ground truth. We on the other hand point out the advantages that having access to different annotations of the same image offer.

Due to the high costs associated with manual labelling, small training sets are ubiquitous and semi-supervised methods, which try to efficiently exploit available unlabelled data, have therefore been widely used in this area [5, 6, 7, 8].

We argue that disagreeing annotators provide us with the opportunity to estimate the aleatoric uncertainty [9] directly from the data and we demonstrate on two public data sets how this uncertainty estimate can efficiently improve pseudo-labelling approaches [10].

### 1.1 Semi-supervised literature

Semi-supervised methods for deep learning are dominated by two approaches: consistency regularisation and entropy minimisation. Consistency regularisation methods aim to use the unlabelled data to accelerate feature learning by training the model to predict the same output for the input under small perturbations. Prominent examples include the Π-model [5] and its extensions like temporal ensembling [6] or mean-teacher models [7].

The alternative approach to semi-supervised learning is entropy minimisation [11] and pseudo labelling [10]. While entropy minimisation encourages the model to make confident predictions regardless of the class, the pseudo labelling approach targets the model’s own predictions if its confidence is higher than some threshold. However, even confident predictions might be wrong which can mislead the model and consequently degrade its performance. This prompted researchers to investigate other metrics besides the confidence to judge the reliability of the model predictions and it was shown that incorporating the uncertainty of the predictions into the selection of pseudo labels [12, 13] allows to improve the performance to the same level as consistency regularisation approaches.

### 1.2 Uncertainty estimation

The uncertainty involved in the model’s predictions can be divided into epistemic uncertainty which refers to the model parameters and stems from the finite sample size of the training process and aleatoric uncertainty which is inherent to the data and cannot be eliminated [9].

Several approaches have been proposed to estimate these uncertainties: MC dropout [14] uses several forward passes through the model with different drop-out conditions to estimate the model uncertainty by considering the variation in the different predictions. Other methods include combining the predictions from an ensemble of models [15] trained with different initial states or using models trained with different weights for classification errors [16]. The aleatoric uncertainty has been tried to be captured by combining a U-Net with a variational autoencoder for a generative model [17] or by placing a Normal prior on the data distribution [18].

### 1.3 Multiple annotators

Due to the low signal-to-noise ratio commonly found in biomedical images, conflicting annotations even by experts are common and pose a challenge to segmentation methods as there is no clear optimal way to combine different annotations into one ground truth in the training process [1]. Several approaches [19, 2, 3] try to model each annotator and their reliability separately and base their ground truth estimation on this. Another approach is to account for conflicting areas by adopting the loss function to allow for ambiguous regions [4].

However, to the best of our knowledge, no work used conflicting annotators as a source for uncertainty estimation in a semi-supervised setting so far.

## 2 Method

For every labeled image *X*_*i*_ ∈ 𝒳= ℝ^*W* ×*H*^ with *i* = 1, …, *N*, there are *k*_*i*_ different annotations $Y_{i}^{k_{i}}\in Y^{W\times H}$ available where we restrict 𝒴 to be the case of binary labels {0, 1}. Moreover, denote the unlabeled images by *Z*_*j*_ with *j* = 1, …, *M* and our segmentation network by *f*_*θ*_ : 𝒳 → [0, 1]^*W* ×*H*^ predicting a probability of belonging to the positive class for every pixel.

Basic pseudo labelling approaches make a prediction ${\tilde{y}}_{j}=f_{\theta }\left(Z_{j}\right)$ for every unlabelled image and restricts itself to the pixels predicted with a high confidence in either the positive or negative class with a mask given by ${ℳ}_{j}=𝟙\left[{\tilde{y}}_{j}>\tau_{pos\;}\right]+𝟙\left[{\tilde{y}}_{j}<\tau_{neg\;}\right]$ based on some thresholds *τ*_*pos*_ and *τ*_*neg*_. Denote the restricted predictions by $f_{\theta }^{ℳ}\left(Z_{j}\right)=ℳ⊙f_{\theta }\left(Z_{j}\right)$ and the restricted pseudo labels by ${\hat{Y}}_{j}^{ℳ}=ℳ⊙𝟙\left[{\tilde{y}}_{j}>0.5\right]$ where ⊙ denotes the element-wise multiplication. Given some loss function *l* (e.g. the binary cross entropy), the training procedure in iteration *t* now takes the form of minimizing

$$ℒ(\theta )=\frac{1}{N}\underset{i}{\sum }l\left(f_{\theta }\left(X_{i}\right),{\bar{Y}}_{i}\right)+\frac{\lambda (t)}{M}\cdot \underset{j}{\sum }l\left(f_{\theta }^{ℳ}\left(Z_{j}\right),{\hat{Y}}_{j}^{ℳ}\right)$$

where ${\bar{Y}}_{i}=s\left({\left\{Y_{i}^{j}\right\}}_{j=1}^{k_{i}}\right)$ is some ground truth aggregation like majority voting and *λ*(*t*) is some time dependent scaling factor.

Similar to the approach developed by Rizve and colleagues [12], we propose to base the pseudo-label mask ℳ_*j*_ not only on the confidence of the model but also on the uncertainty of the prediction. In order to estimate the uncertainty the dominant approach is to use MC-Dropout or Bayesian networks and it has been shown that this allows to improve the segmentation performance. However, while Bayesian networks are in general computationally very hard to train, MC-Dropout requires numerous forward passes for every sample to estimate the uncertainty which induces a high computational overhead. Alternatives like ensemble methods on the other hand require both training a large number of networks as well as forward passes through each of them for every prediction. Additionally, there is still debate how well these methods capture the true underlying uncertainty [20].

We therefore propose to estimate the aleatoric uncertainty directly from the annotator disagreement which allows to use all the available information contained in the mulitple annotations efficiently. Moreover, it avoids the computational over-head of the alternative methods described above.

**Fig. 1:**
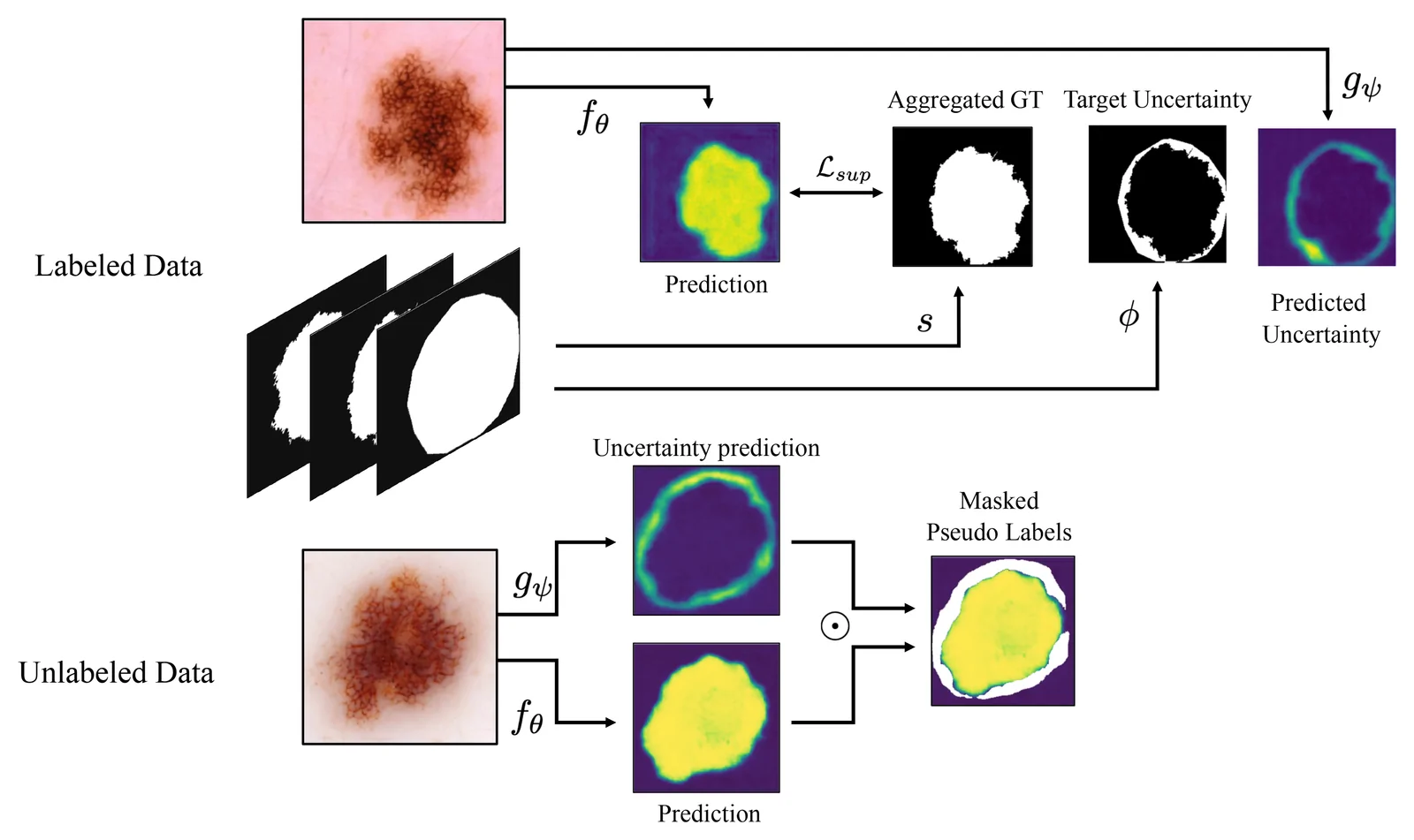
The labelled data is used both to train the uncertainty detection network *g*_*ψ*_ and for the supervised part of the loss function of the training of the segmentation network *f*_*θ*_. Moreover, for the unlabelled data we create both a segmentation and uncertainty prediction but only use the areas of low uncertainty for the pseudo labelling step.

Our approach is summarised in Figure 1. In order to estimate the aleatoric uncertainty, we propose to combine the available annotations for each image into an uncertainty measure *ϕ* : 𝒴 × … × 𝒴 → [0, 1]^*W* ×*H*^. Examples for *ϕ* include a binary indicator for annotator disagreement per pixels or the normalised standard error of annotations per pixel. Subsequently, we train a separate network *g*_*ψ*_ : 𝒳 → [0, 1]^*W* ×*H*^ on the available labelled data that aims to predict the value of *ϕ* at the pixel level using standard supervised techniques. In the training process of *f*_*θ*_, the trained uncertainty network *g*_*ψ*_ predicts for each unlabelled image which areas are likely to cause annotators to disagree and therefore indicate a high level of aleatoric uncertainty. This uncertainty estimate is subsequently used to further refine the pseudo label mask from the prediction confidence by setting $\bar{ℳ}=𝟙\left[g_{\psi }\left(Z_{i}\right)<\tau_{cert\;}\right]⊙ℳ$ based on some threshold value *τ*_*cert*_ and ℒ (*θ*) is minimized.

## 3 Experiments

### 3.1 Data

Our experiments are based on two public datasets which both include multiple annotations per image. The first is the 2018 challenge of the International Skin Imaging Collaboration (ISIC) [21] containing color dermoscopy images coming with up to five annotations per image. For this dataset, a considerable inter-annotator variability has been reported [22]. As we are interested in a semi-supervised setting, we will limited the available training data to 1125 images which have at least 2 annotations associated with them and use the remaining data for validation (250) and testing (1200).

Secondly, we explore the MRI prostate segmentation data from the 2021 QUIBIQ challenge [23] which provides six annotations per image and we use 43 images for training, 5 for validation and 7 for testing (which corresponds to their provided validation set).

All data is resized to 256 × 256 pixels and normalised to [0, 1]. To simulate the semi-supervised setting, we keep 10% from the training data set as labelled and consider the remaining images as unlabelled by disregarding their annotations.

### 3.2 Implementation Details

In order to focus our experiments on the different sources of uncertainty, we decided to use a simple U-Net architecture for both the segmentation network *f*_*θ*_ as well as the uncertainty detection network *g*_*ψ*_ and we incorporate dropout layers for the MC-Dropout experiments. In order to aggregate the different annotations for the supervised training and evaluation we consider the majority vote between annotators as the aggregation function *s*. Moreover, we use the standard deviation for the uncertainty function *ψ* and the binary cross entropy for the supervised loss *l*. For the function scaling the influence of the semi-supervised loss we choose *λ*(*t*) = exp(− (1 − *t/*3000)*/*0.2). Moreover, we use the Adam optimizer with a learning rate of 10^−5^ for both data sets. Finally, we train every model until there is no further decrease in the validation loss for 50 epochs and then chose the model best performing on the validation set. For the evaluation, we compute the intersection-over-union (IoU) and the Dice coefficient.^2^

### 3.3 Results

To judge the effectiveness of using the inter-annotator disagreements as an indication for the aleatoric uncertainty, we first establish a baseline of a fully supervised approach and ignore the unlabelled data. Moreover, we include the results of a simple pseudo-labelling strategy by solely basing the selection of the pseudo labels on the model confidence similar to [10]. Additionally, we compare against the approach suggested by [12] by including the uncertainty measure through eight separate forward passes of a drop-out U-Net and an ensemble of networks. For the ensemble, we first use the available labelled data to train six separate networks with different initial conditions and data orderings as suggested by [15]. After that a final model as described in section 2 is trained with the uncertainty based on the disagreement of the ensemble models.

As it can be seen in Table 1, using our approach allows the pseudo labelling approach to make the biggest increase in performance for both data sets. Additionally, training it is roughly 3 times faster compared to the MC-Dropout method per training iteration and it does not require the additional training of a full ensemble.

Moreover, we investigate the claim that our model provides more reliable pseudo labels compared to the confidence approach alone. Since we are in an experimental setting, we actually have access to the annotations of the data which we consider as unlabelled and are able to compare our masked pseudo labels to the annotations. For the ISIC data, we find that the precision of the predictions on the unlabelled data rose from 0.64 for the confidence based pseudo labels to 0.73 if we include our uncertainty predictions. For the QUBIQ data, we observe an increase from 0.71 to 0.75.

## 4 Conclusion

In this paper we have shown that multiple annotators per sample provide valuable information about the uncertainty in the data and we demonstrated how this uncertainty estimate can be used to improve semi-supervised segmentation techniques by excluding unreliable pseudo-labels. Additionally, our experimental results confirm that our approach improves the resulting segmentation compared to competing approaches and that it reduces the computational complexity of the uncertainty estimation.

However, this paper should not be considered as an alternative to existing methods which try to extract the underlying ground truth from multiple annotations: while we have used a simple majority vote in our experiments to aggregate different labels for the supervised part, it is straightforward to integrate more sophisticated techniques and combine them with our approach. Similarly, we intentionally performed our analysis using a standard U-Net as a well studied baseline model but it is evident that more advanced segmentation models could be utilised to further improve performance.

We are confident that the presented approach can prove valuable to practitioners in medical settings where data from multiple annotators is available in order to integrate every expert’s opinion and as a way to exploit unlabelled data that would go unused otherwise.

## Figures and Tables

**Fig. 2:**
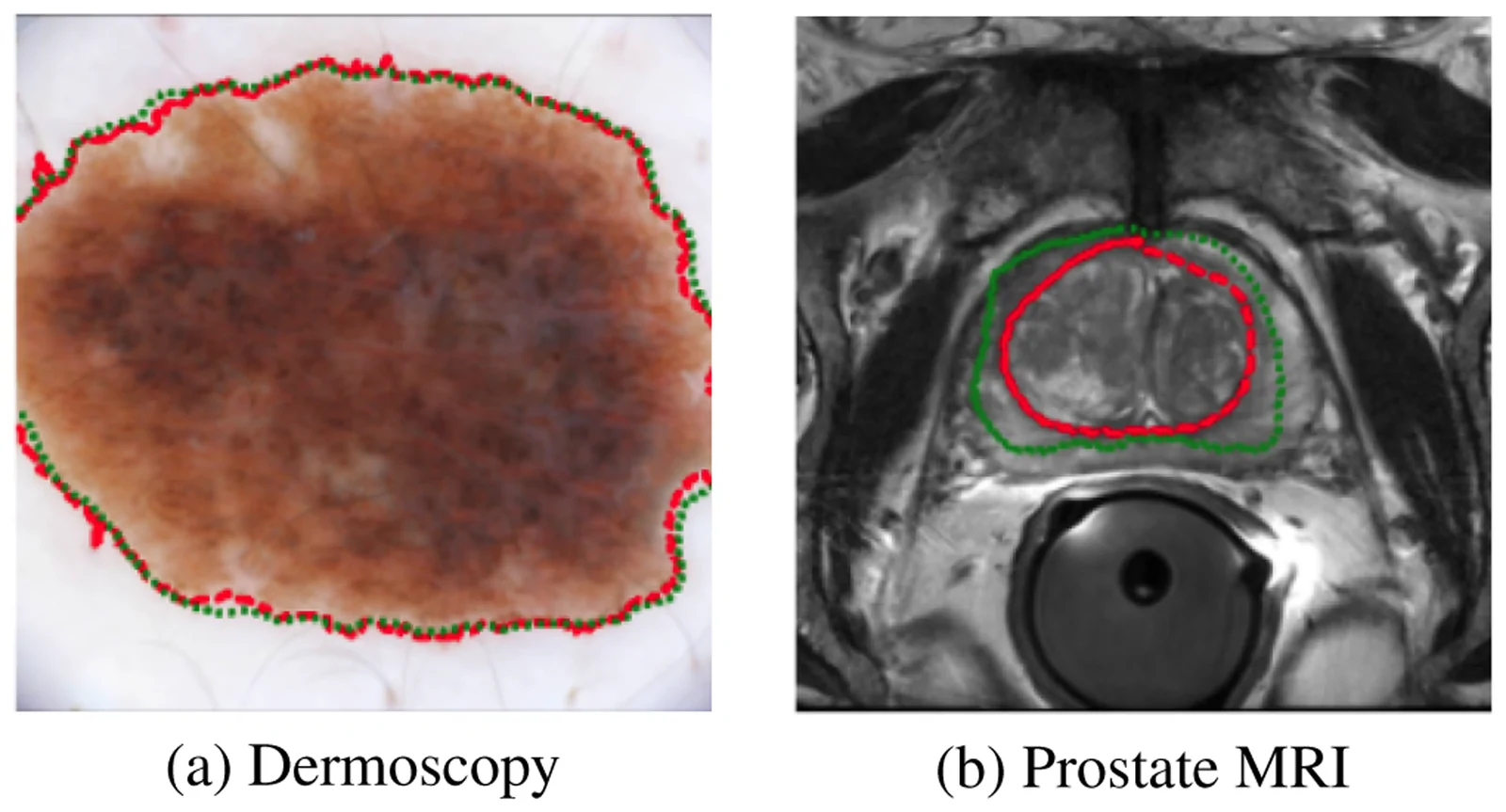
Example dermoscopy taken from ISIC (a) and prostate MRI form QUBIQ prostate (b) dataset with the majority vote in red and our prediction in green.

**Table 1:** Comparison of different pseudo-labeling approaches showing that our approach outperforms several alternatives.

Model	ISIC [21]		QUBIQ [23]	
	IoU	Dice	IoU	Dice
Purely Supervised	0.67	0.77	0.34	0.45
Pseudo Labels [10]	0.69	0.78	0.46	0.57
MC-Dropout [12]	0.70	0.79	0.46	0.58
Ensemble [15]	0.72	0.81	0.52	0.63
Ours	**0.74**	**0.83**	**0.54**	**0.66**

## Footnotes

2 The code will be made available on github with the final submission.
